# Two subspecies of bent-winged bats (*Miniopterus orianae bassanii *and *oceanensis*) in southern Australia have diverse fungal skin flora but not *Pseudogymnoascus destructans*

**DOI:** 10.1371/journal.pone.0204282

**Published:** 2018-10-10

**Authors:** Peter H. Holz, Linda F. Lumsden, Marc S. Marenda, Glenn F. Browning, Jasmin Hufschmid

**Affiliations:** 1 Department of Veterinary Biosciences, Melbourne Veterinary School, The Faculty of Veterinary and Agricultural Sciences, The University of Melbourne, Werribee, Victoria, Australia; 2 Asia-Pacific Centre for Animal Health, Melbourne Veterinary School, The Faculty of Veterinary and Agricultural Sciences, The University of Melbourne, Parkville, Victoria, Australia; 3 Arthur Rylah Institute for Environmental Research, Department of Environment, Land, Water and Planning, Heidelberg, Victoria, Australia; CSIRO, AUSTRALIA

## Abstract

Fungi are increasingly being documented as causing disease in a wide range of faunal species, including *Pseudogymnoascus destructans*, the fungus responsible for white nose syndrome which is having a devastating impact on bats in North America. The population size of the Australian southern bent-winged bat (*Miniopterus orianae bassanii*), a critically endangered subspecies, has declined over the past 50 years. As part of a larger study to determine whether disease could be a contributing factor to this decline, southern bent-winged bats were tested for the presence of a range of potentially pathogenic fungi: *P*. *destructans*, dermatophytes and *Histoplasma capsulatum* (a potential human pathogen commonly associated with caves inhabited by bats). Results were compared with those obtained for the more common eastern bent-winged bat (*M*. *orianae oceanensis*). All bats and their environment were negative for *P*. *destructans*. A large number of fungi were found on the skin and fur of bats, most of which were environmental or plant associated, and none of which were likely to be of significant pathogenicity for bats. A 0–19% prevalence of *H*. *capsulatum* was detected in the bat populations sampled, but not in the environment, indicative of a low zoonotic risk. Based on the results of this study, fungi are unlikely to be contributing significantly to the population decline of the southern bent-winged bat.

## Introduction

It has long been recognized that fungi can have detrimental effects on agricultural crops and wild flora [1]. However, an increasing number of fungi are being documented as animal pathogens causing disease across a wide range of species, including *Aspergillus sydowii* in corals, *Nosema* sp. in bees and *Cryptococcus gattii* in humans and wildlife [2]. Two recently emerged fungal pathogens have caused widespread declines in their host populations: *Batrachochytrium dendrobatidis*, which causes chytridiomycosis in amphibians, and *Pseudogymnoascus destructans*, which causes white nose syndrome (WNS) in bats [3].

White nose syndrome, caused by the psychrophilic fungus, *Pseudogymnoascus destructans*, has killed millions of bats in North America since its appearance there in 2006 [4]. It is believed to have spread from Europe, where it appears to cause only minimal disease [5]. To date, no cases of WNS have been diagnosed in Australia, and the fungus is presumed to be absent. A wild bat from Queensland with a histologically identifiable infiltrative fungal dermatitis on the face and wings was tested for *P*. *destructans*, but found to be negative [6]. Environmental conditions in most Australian bat populations are probably not conducive to the catastrophic spread of disease observed in North American populations, because of the low temperatures required by the fungus to thrive and the need for high densities of bats to maintain very close contact for transmission, as occurs in hibernating cave-dwelling bats in North America [7]. However, bent-winged bat populations could potentially fulfil the conditions needed for a catastrophic outbreak, as they hibernate in high densities within caves that remain below 20°C throughout winter [8]. A recently completed risk assessment concluded that the southern bent-winged bat population may be at significant risk should the fungus enter Australia [8].

As well as WNS, bats may also carry other potentially pathogenic fungi. However, only a few studies have investigated this in any detail [9–14]. A recent survey of skin diseases of captive North American, European and Australasian bats identified a range of lesions but, while some bacterial cultures were performed, fungal causes were not explored [15]. There is very little knowledge about which skin-related fungi occur on Australian bats, or whether any of them have the potential to cause outbreaks affecting significant proportions of bat colonies or populations, or whether they affect survival rates.

Some bat-associated fungi are known to be zoonotic. *Histoplasma capsulatum* is a dimorphic fungus that grows as a mould at 25°C and a yeast at 37°C [16]. It is a potential human pathogen associated with large amounts of guano, as can be found in caves that house bats and chicken coops. While the majority of human infections are asymptomatic, the fungus can cause pulmonary disease, oropharyngeal ulceration and septicaemia [16, 17]. *Histoplasma capsulatum* is found in temperate areas throughout the world, including Australia [17–19]. It prefers a high nitrogen substrate, a temperature range between 22–29°C and a relative humidity of 68–90%, conditions frequently found in caves [19]. Infections are usually acquired by inhalation, but can also be contracted through ingestion or by skin contact, but not by direct transmission from infected humans [20]. The fungus can be found in soil and guano and is carried by bats, having been isolated from their lungs, spleen, liver and gastrointestinal tract [21, 22]. While *H*. *capsulatum* has been associated with disease in a range of hosts, including dogs, cats, horses, primates, mustelids, procyonids [23], reindeer (*Rangifer tarandus*) [24] and mara (*Dolichotis patagonum*) [25], bats are usually asymptomatic, although experimental infections have resulted in morbidity and mortality [21]. *Histoplasma capsulatum* was selected for more detailed study, not because of its possible pathogenicity for bent-winged bats, but because of the potential to affect the humans working with these animals.

The Australian bent-winged bats are small, cave-roosting, insectivorous bats, weighing around 15 g [26]. There are two subspecies of large bent-winged bat (*Miniopterus orianae*) in south-eastern Australia that form separate maternity colonies (Cardinal and Christidis 2000). The southern bent-winged bat (*M*. *orianae bassanii*) occurs only in south-western Victoria and south-eastern South Australia (SA). There are three main maternity caves, one near Warrnambool (38.3687° S, 142.4982° E), one near Cape Bridgewater (38.3013° S, 141.4062° E) both in Victoria and the other near Naracoorte (36.9602° S, 140.7413° E) in SA [27]. The size of the populations centred on these two maternity caves has declined dramatically over the last 50 years [27], and the subspecies was listed as critically endangered in 2007 under the *Commonwealth Environment Protection and Biodiversity Conservation Act 1999*. The eastern bent-winged bat (*M*. *orianae oceanensis*) is more common and widespread, being distributed along the east coast of Australia [27]. Although numbers appear to be stable, it is listed as vulnerable in Victoria due to the dependence on just one maternity cave.

The aim of this study was to test southern and eastern bent-winged bats for exposure to a range of potentially pathogenic and/or zoonotic fungi: *P*. *destructans*, dermatophytes and *H*. *capsulatum*. Not detecting *P*. *destructans* would strengthen the case that Australia is currently free of this fungus facilitating the implementation of stricter biosecurity measures for visitors to caves housing bats.

## Materials and methods

### Study population and sites

Southern and eastern bent-winged bats were captured outside cave entrances at a number of different locations in Victoria and South Australia between April 2015 and August 2017. Access to the main maternity cave of southern bent-winged bats in Victoria was technically very restrictive with only opportunistic sampling possible. Therefore, most southern bent-winged bats were trapped at nearby caves, identified in this study by their closest towns: Allansford (38.3861° S, 142.5931° E) and two caves near Portland (38.3609° S, 141.6041° E). South Australian southern bent-winged bats were trapped at the breeding cave entrance near Naracoorte. Eastern bent-winged bats were trapped at the entrances of disused mines at Christmas Hills (37.6515° S, 145.3173° E) and Eildon (37.2343° S, 145.8976° E) in central Victoria and at the Victorian maternity cave near Lakes Entrance (37.8511° S, 147.9958° E) in eastern Victoria. Sampling periods were chosen when cave conditions were most conducive to fungal growth, to optimise detection of *P*. *destructans* (Table 1). The average temperature in the Victorian breeding cave in September is 11°C with a relative humidity of 84%, while the mean Naracoorte cave temperature is 16°C with a relative humidity of 60%. In North America and Europe greater loads of *P*. *destructans* are present on bats towards the end of winter, thereby increasing the possibility of detecting the fungus at this time [4, 28, 29]. However, sampling of bats for other diseases [30] did occur in the middle of summer and early autumn. Therefore, samples collected in January, February and March were tested for dermatophytes and *H*. *capsulatum*, but not *P*. *destructans*. Sampling did not occur in the middle of winter, to avoid disturbance to hibernating bats, which could have significant negative impacts on the populations [31].

**Table 1:** Sampling sites, dates and prevalence of *Pseudogymnoascus destructans*, *Histoplasma capsulatum* and skin-associated fungi in southern and eastern bent winged bats surveyed in Victoria and South Australia. n = sample size.

Location	Date	n	*P*. *destructans (%)*	*H*. *capsulatum* DNA (%)	Fungal DNA (%)
**Southern bent-winged bats**					
Warrnambool	September 2015	6	0*	0	NT
Allansford	September 2015	32	0	13	75
Portland 1	September 2016	45	0	0	16
Portland 2	February 2017	44	NT	14	80
Portland 2	August 2017	67	0	NT	NT
Naracoorte	January 2016	37	NT	19	8
Naracoorte	September 2016	76	0	13	52**
**Eastern bent-winged bats**					
Christmas Hills	April 2015	35	0	0	100
Christmas Hills	September 2015	26	0	4	92
Eildon	September 2016	39	0	15	13
Lakes Entrance	March 2017	51	NT	6	24

NT = Not tested

*n = 5

**n = 75

### Sample collection and laboratory processing

Individuals were caught as they flew out of the caves/mines, using modified harp traps (Austbat, Bairnsdale, Victoria [32]) set at dusk at the entrances. Two harp traps were used at each cave/mine, with the exception of the Naracoorte cave, which had a more exposed entrance necessitating the use of 12 traps. Traps were monitored continually, with the bats either left in the harp trap bag, or transferred in small numbers (a maximum of 10 per bag) to cloth bags, prior to sampling.

All bats were examined for any signs of disease, aged as juveniles or adults (based on the presence of a cartilaginous core at the metacarpal-phalangeal joint [33]), sexed, weighed, and forearm measured from carpus to elbow.

Sterile, rayon tipped, cotton swabs (Copan Flock Technologies, Brescia, Italy) were dipped in sterile saline and then a rolling action was used to wipe several long strokes across both ventral and dorsal wing surfaces and around the muzzles of all bats that were trapped.

Two separate swabs were used for each bat. One swab was used for *H*. *capsulatum* detection. After sampling, this swab was placed in Sabouraud’s broth (Sigma-Aldrich, Castle Hill, NSW) and kept at 4°C until it was transported to the laboratory.

The other swab was used to test for *P*. *destructans*. For the first three sampling trips (Christmas Hills 1 and 2, Allansford), the swab was placed back into its sleeve after sampling and held at 4°C until it was transported to the laboratory, where it was stored at -20°C until assayed. These swabs were also used for dermatophyte testing. However, there was some concern that, as no clinical cases of WNS had been observed in Australia, *P*. *destructans*, if present, could be at very low concentrations on bats, potentially decreasing the likelihood of detecting the fungus on a dry swab by using this protocol. To increase the likelihood of detection, swabs collected on subsequent trips (Portland 1, Naracoorte September 2016, Portland 2 August 2017 and Eildon) were placed in Sabouraud’s broth and incubated at 4–6°C for two to four weeks prior to testing. A similar technique was used in a Swedish survey for *P*. *destructans* [34]. Dermatophyte testing was subsequently performed on the swabs collected for surveillance for *H*. *capsulatum*.

All bats were examined for clinical signs of WNS using a 12 x LED ultraviolet torch emitting ultraviolet light with a wavelength of 365 to 375 nm (The LED Shop, Herberton, Australia), as this has been shown to be an accurate way to detect lesions that may otherwise have not been detected [35].

As part of a larger study, oral swabs were also collected for examination for viruses [30], blood was collected for haematological and biochemical analyses, and ectoparasites were collected. Following sampling, all bats were released at the point of capture, typically within four to six hours.

As *P*. *destructans* has been shown to persist in the environment [36, 37], 30 swab samples were collected from guano and cave surfaces. A 100 metre line was walked through the Naracoorte cave, and the environment was swabbed at 10 metre intervals. For the other caves, swabs were taken from the ground under roosting bats. In addition to the caves from which bats were trapped, a second cave near Allansford and one at Cape Bridgewater (38.3013° S, 141.4062° E) were sampled. Swabs were dipped into guano piles or wiped over the rock surfaces of the caves. All swabs were then kept at 4°C until transported to the laboratory, where they were stored at -20°C until assayed. These samples were collected before the incubation method was initiated and were processed without incubation.

As *H*. *capsulatum* has been associated with guano and cave surfaces [22], 51 samples were collected from caves inhabited by bent-winged bats. In addition to swabs from the caves sampled for *P*. *destructans*, swabs were also collected from caves at Byaduk (37.9352° S, 141.9653° E) and Mt. Porndon (38.3128° S, 143.2878° E), and from the interior of the Warrnambool maternity cave. The probability of detecting *H*. *capsulatum* has been shown to be greater if guano is sampled below the surface [19], so swabs were dipped into guano piles (rather than collecting from the surface of the guano), or wiped over the rock and soil surfaces. As the fungus has been shown to be unevenly distributed throughout a cave [21, 22], it was thought possible that random sampling of cave surfaces may not accurately reflect the presence or absence of the fungus. Therefore, swabs were also taken of the sides and soles of the boots worn by three participants following a trip to the Warrnambool maternity cave.

### Necropsies

At the start of the study an initial visit to the Warrnambool cave revealed some dead, decomposed bat remnants scattered within the cave. The remnants consisted of fur, bones and wing fragments, and appeared to be clustered together in several groups under ledges around the periphery of the cave. The groups contained 26, 11, 11 and one bat remnants. Over the course of the study, four juvenile bats from the Naracoorte cave were euthanized because of fractures incurred while flying out of the cave (unrelated to capture), and three bats were found dead from unknown causes (two from the Naracoorte cave and one from the Warrnambool cave). The skin of these bats and bat remnants was swabbed and tested for *P*. *destructans* and *H*. *capsulatum*. Following a complete necropsy of the seven freshly dead bats, swabs were wiped through the abdominal cavity and over the internal organs. The swabs were then placed in Sabouraud’s broth for *H*. *capsulatum* testing.

**Fig 1:**
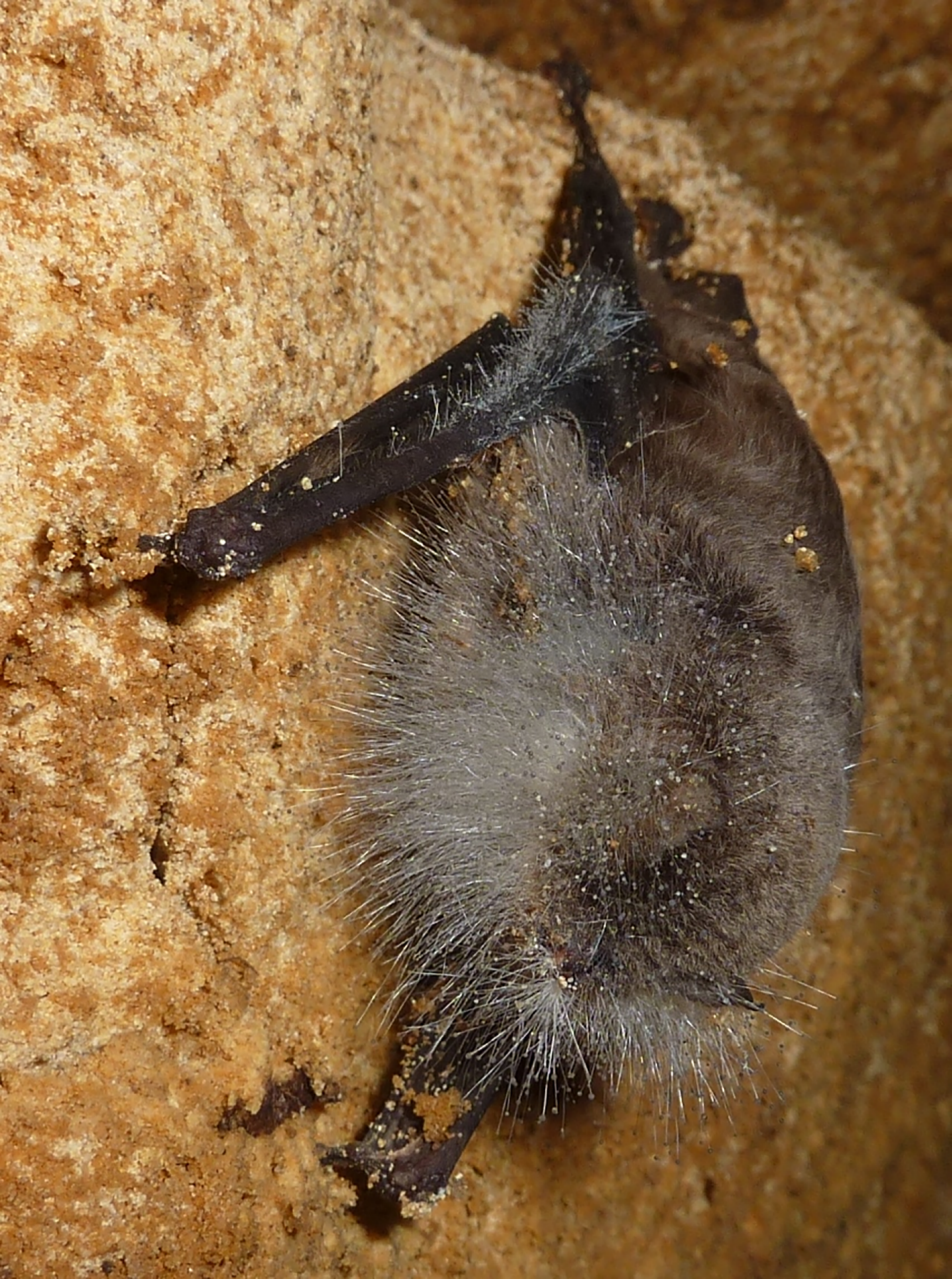
Photograph of the dead southern bent-winged bat covered in white fungal material that was found in Portland 2 cave (‘UBat’).

A single dead southern bent-winged bat, which appeared to be covered in white fungal material (termed ‘UBat’), was found in Portland 2 cave in October 2016 (Fig 1). A swab was taken from this bat and DNA extracted and tested for *P*. *destructans*.

### Air sampling

To test for the possibility of exposure to *H*. *capsulatum* by aerosol, an air sampler (Coriolis Micro, Bertin Instruments, France) was taken into the Warrnambool cave in September 2016 and air samples were collected within the cave at six locations where large numbers of bats roosted, and there were guano piles and human traffic. The air sampler cone was filled with 15 ml sterile saline and the sampler was run at an air flow rate of 300 L/min for ten minutes at each of the locations. The entire volume of saline was inoculated into Sabouraud’s broth, which was kept at 4°C until it was transported to the laboratory.

All samples were taken to the laboratory within 36 hours of collection.

### Laboratory methods

DNA from the swabs and from 300 μl aliquots of the Sabouraud’s broths to be tested for *P*. *destructans* was manually extracted using the method described by Steer et al. [38]. Extracted DNA was used as a template in a PCR assay, using primers (5′-GGGGACGTCCTAAAGCCT-3′, 5′-TTGTAATGACGCTCGGAC-3′) targeting a 624 bp region of the ribosomal RNA gene internal transcribed spacer (ITS) [39]. PCR negative controls containing no DNA template and PCR positive controls containing extracted *P*. *destructans* DNA, obtained from the Mycology Reference Laboratory, Bristol, UK, were also included. PCR products were visualised after electrophoresis through an agarose gel.

Broths containing swabs to be tested for *H*. *capsulatum* were placed in an incubator at 37°C for 72 hours to allow the growth of the non-infectious yeast form of the fungus. Prior to opening the vials, organisms in the cultures were then inactivated by incubating them in a water bath at 90°C for 10 minutes [40].

Once inactivated, fungal DNA was extracted, using the same method as described for *P*. *destructans*. Extracted DNA was used as a template in a PCR assay, using primers (5′-GCGTTCCGAGCCTTCCACCTCAAC-3′, 5′-ATGTCCCATCGGGCGCCGTGTAGT-3′) designed to amplify a 391 bp region of a gene coding for a 100-kDa-like protein, specific for *H*. *capsulatum* [41]. PCR negative controls containing no DNA template and PCR positive controls containing extracted *H*. *capsulatum* DNA, obtained from the Mycology Reference Laboratory, Bristol, UK, were also included. PCR products were visualised after electrophoresis through an agarose gel and products from a selection of the positive samples were purified (QIAquick Gel Extraction Kit, Qiagen, Melbourne, Australia) and subjected to Sanger sequencing (using Big Dye Terminators version 3.1, Applied Biosystems, Melbourne, Australia) to confirm their identity.

Dermatophyte testing was only undertaken on swabs collected from bats. Extracted DNA was used as a template in a PCR, using primers (5′-TCCGTAGGTGAACCTGCGG-3′, 5′-TCCTCCGCTTATTGATATGC-3′) designed to amplify a 330 bp region of the fungal ribosomal RNA genes ITS1 and ITS4 [42]. PCR products were separated by electrophoresis through an agarose gel.

The most prominent bands on each gel were purified from the agarose (QIAquick Gel Extraction Kit, Qiagen, Melbourne, Australia) for further analysis. DNA was quantified in each sample using a NanoDrop spectrophotometer (Thermo Fisher Scientific, USA) and then submitted to a commercial laboratory (Australian Genome Research Facility, Gehrmann Laboratories, University of Queensland, Australia) for diversity profiling.

PCR amplicons were generated using the ITS1 (CTTGGTCATTTAGAGGAAGTAA) and ITS2 (GCTGCGTTCTTCATCGATGC) primers. PCR cycle conditions were: 7 minutes at 95°C, followed by 35 cycles of 30 seconds at 94°C, 45 seconds at 55°C and 60 seconds at 72°C, followed by a final 7 minute extension at 72°C. Thermocycling was completed with an Applied Biosystem 384 Veriti and using AmpliTaq Gold 360 mastermix (Life Technologies, Australia) for the primary PCR. The first stage PCR was cleaned using magnetic beads, and samples were visualised on 2% Sybr Egel (Thermo Fisher Scientific, USA). A secondary PCR to index the amplicons was performed with TaKaRa Taq DNA Polymerase (Clontech, USA). The resulting amplicons were cleaned again using magnetic beads, quantified by fluorometry (Promega QuantiFluor, Australia) and normalised. The equimolar pool was cleaned a final time using magnetic beads to concentrate the pool and then measured using a High-Sensitivity D1000 Tape on an Agilent 2200 TapeStation. The pool was diluted to 5nM and molarity was confirmed again using a High-Sensitivity D1000 Tape. This was followed by sequencing on an Illumina MiSeq (San Diego, CA, USA) with a V3, 600 cycle kit (2 x 300 base pairs paired-end).

Paired-ends reads were assembled by aligning the forward and reverse reads using PEAR (version 0.9.5) [43]. Primers were identified and trimmed. Trimmed sequences were processed using Quantitative Insights into Microbial Ecology (QIIME 1.8) [44] USEARCH (version 8.0.1623) and UPARSE software [45, 46].

Using USEARCH tools sequences were quality filtered, full length duplicate sequences were removed and sorted by abundance. Singletons or unique reads in the data set were discarded. Sequences were clustered followed by chimera filtered using “Unite” database as reference. To obtain number of reads in each OTU, reads were mapped back to OTUs with a minimum identity of 97%. Using QIIME, taxonomy was assigned using Unite database (Unite Version7.1 Dated: 22.08.2016) [47].

A total of 26 samples collected from bats were submitted, but only 18 of these contained sufficient DNA for profiling. Sequences were deposited in the NCBI database BioProject ID PRJNA484260.

### Statistical analyses

A range of potential internal and external predictor variables were screened for association with detection of *H*. *capsulatum* DNA, using univariable logistic regression. These included subspecies, location (grouped as South Australian southern bent-winged bat, Victorian southern bent-winged bat and Victorian eastern bent-winged bat), body mass, sex, and age (adult or juvenile) (internal factors). Season (spring, summer, autumn) was the only external factor included. All factors significant at P < 0.20 were subsequently included in a multivariable logistic regression model, using backward stepping. The final model only included those variables significant at P < 0.05. All statistical analyses were performed using Minitab 18 (Minitab, USA).

## Results

A total of 325 live bats and 30 environmental samples were tested by PCR for the presence of *P*. *destructans*. All samples were negative.

The swab sample taken from the dead southern bent-winged bat that was found covered in white fungal material in Portland 2 cave (‘UBat’) yielded a band on the agarose gel, suggesting the presence of *P*. *destructans*. The DNA sequence of the product had 99% identity with that of *P*. *destructans* (GenBank Accession No. KP714633.1). A sample was submitted to the Australian Animal Health Laboratory (AAHL) in Geelong for confirmation. Further testing by AAHL indicated that this isolate did not contain the C-deletion at position 125 described by Shuey et al. (2014) as indicative of *P*. *destructans*. Therefore, the sample was classified as negative. Diversity profiling found that 66.4% of this bat’s fungal flora consisted of a *Gymnoascus* species (Table 2).

**Table 2:** Fungal diversity profiling results expressed as a percentage of the total mycobiome on 18 bent-winged bats from five caves. Sampling location: CH = Christmas Hills, LE = Lakes Entrance, A = Allansford, N = Naracoorte, P = Portland, Month of sampling: Apr = April, Sep = September, Jan = January, Feb = February, Oct = October, Mar = March. Bat identification number including the sex of the individual: M = Male, F = Female, U = Unknown.

	Eastern bent-winged bat									Southern bent-winged bat								
Location	CH	CH	CH	CH	CH	CH	CH	LE	LE	A	A	A	N	N	N	P	P	P
Month of sampling	Apr	Apr	Apr	Apr	Apr	Sep	Sep	Mar	Mar	Sep	Sep	Sep	Jan	Jan	Jan	Feb	Feb	Oct
Bat ID	M5	M11	M15	F27	M32	F10	F27	F22	M32	M19	F30	M32	F26	M27	M31	M6	M43	UBat
**Ascomycota**																		
*Alternaria* sp.	7.4	6.1	5.9	5.3	2.7													
*Alternaria betae-kenyensis*																	0.8	
*Alternaria kulundii*											5.7							
*Apodospora* sp.						0.9												
*Arachnopeziza aurata*										1.8								
*Ascomycota* sp.	7.2	4.6	4.2	7.0	4.4	4.0	0.7	2.1		0.3	19.9	5.9		9.5	6.0			11.8
*Aspergillus carbonarius*		1.1																
*Aspergillus flavus*													95.4					
*Aspergillus penicillioides*		0.9															4.3	
*Aspergillus piperis*						1.1										94.5		
*Aureobasidium pullulans*		1.1			10.9													
*Bagadiella koalae*		0.7																
*Camarosporium* sp.			2.4															
*Candida athensensis*																5.5		
*Capnobotryella* sp.		0.8																
*Capnodiales* sp.						6.4	1.0					4.1						
*Chrysosporium* sp.																		1.3
*Cladosporium delicatulum*								10.5	0.6									
*Cochliobolus* sp.		0.7																
*Davidiella tassiana*	18.1	15.0	14.6	28.5	8.6	0.7	25.1								9.6			
*Debaryomyces udenii*						11.1	1.8							1.8				
*Didymella exigua*													0.4					
*Dothideomycetes* sp.											4.2	1.6		79.2				
*Eurotiomycetes* sp.						1.3												
*Gymnoascus* sp.																		66.4
*Hypocreales* sp.				2.2														
*Lasiosphaeriaceae* sp.					2.8													
*Leotiomycetes* sp.						0.6												
*Leptosphaeriaceae* sp.	7.0	5.5	5.7	11.6	7.0	5.3												
*Letendraea* sp.						1.2												
*Microascus longirostris*																		3.3
*Mycosphaerella tassiana*										2.2	6.0	21.3						
*Mycosphaerellaceae* sp.						0.8												
*Myriangiales* sp.		1.1								2.0					4.5			
*Myriodontium* sp.																		4.5
*Myrothecium* sp.				0.7														
*Nectriaceae* sp.					1.1													
*Neocatenulostroma microsporum*									0.2									
*Neodevriesia capensis*											1.7							
*Neophaeosphaeria* sp.				0.9														
*Neophysalospora eucalypti*																	64.5	
*Oidiodendron cereale*																	8.6	
*Paraconiothyrium variabile*				1.1			3.6											
*Penicillium bialowiezense*						0.6												
*Penicillium crustosum*				1.1														
*Penicillium polonicum*																		1.9
*Penicillium spinulosum*	0.6											2.4						
*Phaeothecoidea minutispora*		0.8																
*Phoma calidophila*	13.8	1.8	16.1	2.7	6.0	18.9						2.7						
*Pleospora herbarum*		1.7		0.8														
*Pleosporaceae* sp.	3.1	2.7	2.0	2.4		6.7												
*Pleosporales* sp.	4.7	6.0	5.2	5.1	2.6					1.1	25.2			3.9			7.7	
*Podospora glutinans*					26.1													
*Pseudogymnoascus roseus*	0.03																	
*Readeriella angustia*			1.6															
*Schizothecium carpinicola*					2.5													
*Schizothecium* sp.					3.8													
*Simplicillium aogashimaense*				0.7														
*Sordariomycetes* sp.				0.6									0.1					
*Strelitziana albiziae*												1.5						
*Teratosphaeria capensis*	3.2	2.1	4.17	3.0	2.0													
*Teratosphaeriaceae* sp.															50.7			
*Toxicocladosporium strelitziae*				0.6														
*Trichocomaceae* sp.							0.7											
*Ulocladium chartarum*								86.5										
*Wickerhamia fluorescens*			0.7			2.4												
*Xenophacidiella pseudocatenata*						2.7	0.9											
**Basidiomycota**																		
*Agaricales* sp.			0.8															
*Agaricomycetes* sp.		0.7	1.3															
*Agaricostilbum hyphaenes*			0.6															
*Atheliaceae* sp.												8.6						
*Auricularia mesenterica*				1.7														
*Bandoniozyma noutii*				0.6														
*Basidiomycota* sp.						1.5												
*Brevicellicium olivascens*												21.1						
*Bullera* sp. VY 86			1.6	1.2														
*Bullera* sp.											1.6	6.8						
*Cryptococcus aff* amylolyticus AS 22501						0.7												
*Cryptococcus albidus*	2.7	0.6	6.0	2.3	2.1													
*Cryptococcus friedmannii*												12.0						
*Cryptococcus laurentii*			1.1															
*Cryptococcus* sp.				0.6														
Cryptococcus victoriae*										0.5								
*Cystofilobasidium capitatum*										4.7								
*Duportella* sp. JK 2014															3.7			
*Duportella* sp.																	14.2	
*Gloeocystidiellum* sp.				1.0		1.9												
*Hannaella luteola*															6.1			
*Kondoa aeria*		22.2																
*Limonomyces* sp.						2.6												
*Lycoperdon utriforme*															10.0			
*Ossicaulis lachnopus*											2.0							
*Peniophoraceae* sp.				0.8														
*Polyporales* sp.			0.9															
*Polyporus tricholoma*						0.7												
*Pyrofomes demidoffii*						0.6												
*Rhodotorula mucilaginosa*	1.4	1.3	1.8			2.4				85.9	5.6	0.7	3.9		3.6			
*Rhodotorula slooffiae*	0.7					0.6												
*Rhodotorula taiwanensis*														5.6				
*Sporidiobolales* sp.	6.8	1.1	3.8	1.1	1.6	1.4												
*Sporobolomyces phyllomatis*												1.0						
*Sporobolomyces roseus*									99.1			2.7						
*Sporobolomyces ruberrimus*			0.8															
*Trametes versicolor*						3.4												
*Tremella indecorata*		0.9																
*Tremellomycetes* sp.						1.2												
*Trichosporon cutaneum^*											1.6	1.7						
*Trichosporon guehoae#*		0.8																
*Tubulicrinis* sp.		0.6				1.7												
*Udeniomyces puniceus*	4.0	0.7	3.4	3.9	1.7	2.5					4.3	1.2						
**Zygomycota**																		
*Mortierella indohii*																		9.6
*Mortierella parvispora*				0.7							4.2							
*Mortierella sp*.						1.5												
**Unclassified**																		
Fungi sp.	1.4			0.9	0.9	8.1	61.0				3.5				5.2			

* Now *Vishniacozyma victoriae*.

^ Now *Cutaneotrichosporon cutaneum*.

# Now *Cutaneotrichosporon guehoae*.

A swab of guano from Portland 1 in August 2017 also yielded a band suggestive of the presence of *P*. *destructans*. The sequence of this product had 94% identity with *P*. *destructans*. Consequently, this sample was also submitted to AAHL for confirmation, but yielded a negative result when subjected to the PCR assay described by Shuey et al. (2014).

Between 0 and 19% of bats, depending on location and sampling date, were carrying *H*. *capsulatum* based on PCR (Table 1). The highest proportion of infected bats was found among the southern bent-winged bats from Naracoorte in January 2016 (19%; n = 37), while no *H*. *capsulatum* was detected on southern bent-winged bats from Portland 1 in September 2016 (n = 45) or eastern bent-winged bats from Christmas Hills in April 2015 (n = 35). However, the bats from Christmas Hills did have a low level of *H*. *capsulatum* carriage (4%; n = 26) in September of the same year. All 51 environmental samples were negative, as were the samples from the decomposed bat remnants and necropsied bats.

The prevalence of fungal carriage by the bats, based on PCR, ranged from 8–100% (Table 1). The highest proportion of bats with detectable fungus was among the eastern bent-winged bats from Christmas Hills in April 2015 (100%; n = 35), with this population also having a high prevalence when resampled in September of the same year (92%; n = 26). The lowest prevalence of fungi was among the southern bent-winged bats at Naracoorte in January 2016 (8%; n = 37). This had increased to 52% (n = 75) when the population was resampled in September of that year.

A total of 115 fungal species were identified through the diversity profiling carried out on the 18 bats with sufficient fungal DNA (Table 2). Of these, 80 species were found on eastern bent-winged bats, 39 species on Victorian southern bent-winged bats and 16 species on South Australian southern bent-winged bats. Because of the large number of fungal species identified, fungi constituting less than 0.2% of a bat’s total fungal population were excluded from the table. The only fungal species of interest (because of its close relationship to *P*. *destructans*) present at a prevalence of below 0.2% was *Pseudogymnoascus roseus*, which was found on bat ‘M5’ at a prevalence of 0.03%.

The most commonly occurring fungal species were *Rhodotorula mucilaginosa* (found on four eastern bent-winged bats and five southern bent-winged bats), *Pleosporales* sp. (five eastern bent-winged bats and four southern bent-winged bats), *Davidiella tassiana* (seven eastern bent-winged bats and one southern bent-winged bat), *Udeniomyces puniceus* (six eastern bent-winged bats and two southern bent-winged bats) and *Phoma calidophila* (six eastern bent-winged bats and one southern bent-winged bat).

The largest number of bats with fungi, and the greatest fungal diversity, was found on eastern bent-winged bats from Christmas Hills, which carried 74 of the 115 species identified. *Leptosphaeriaceae* sp. *Pleosporaceae* sp., *Teratosphaeria capensis*, *Cryptococcus albidus* and *Sporidiobolales* sp. were only found on Christmas Hills bats. While many of the individual bats of both subspecies carried a number of different fungi, the mycobiome of several bats was dominated by a single fungal species: *Aspergillus flavus* on southern bent-winged bat number ‘F26’ (95.4% of all fungi on the bat), *Aspergillus piperis* on southern bent-winged bat number ‘M6’ (94.5%), *Rhodotorula mucilaginosa* on southern bent-winged bat number ‘M19’ (85.9%), *Sporobolomyces roseus* on eastern bent-winged bat number ‘M32’ (99.1%), and *Ulocladium chartarum* on eastern bent-winged bat number ‘F22’ (86.5%).

Using univariable logistic regression, season was the only significant predictor (i.e. p<0.05) associated with carriage of *H*. *capsulatum* (p = 0.018). This was due to an overall higher probability of finding *H*. *capsulatum* in summer compared to autumn (OR = 6.5; 95% CI = 1.3, 32.5). When placed in a multivariable logistic regression model no predictors had a significant association with carriage. The association between season and the presence of *H*. *capsulatum* was no longer significant when location group was added in the model.

## Discussion

This study is the first survey of the fungal flora of Australian bent-winged bats.

*Pseudogymnoascus destructans* was not detected on any of the bats sampled, nor in their environment. To date, there is only one published survey for *P*. *destructans* on *M*. *schreibersii* (a closely related species) from Europe [48]. In that study, depending on the method used (histopathology, UV light or PCR), the prevalence of carriage was 17, 47 or 100%, respectively. When established in bat populations, the prevalence of *P*. *destructans* in late winter/early spring (when the survey described here was conducted) typically ranges from 4 to 55% when using histology [49], and 25 to 100% when using PCR [48]. A survey of clinically unaffected bats in Tennessee (a region of the USA with a climate more similar to that of south-eastern Australia than that of Europe) found a prevalence of carriage of *P*. *destructans* of 29% [50]. Based on this level of prevalence, only 15 bats would need to be sampled from a population of 10,000 to be 99% confident that the fungus is absent [51]. However, a false negative result might be plausible if the fungus had arrived very recently in one or more of the bat populations surveyed. A North American survey found that the prevalence of *P*. *destructans* increased rapidly on introduction, from 1/129 bats sampled to 100% two years later [52].

While Sabouraud’s broth has been shown to be an effective growth medium for the culture of *P*. *destructans* [53] dextrose-peptone-yeast extract agar produces more viable colonies [54]. However, as this study was not concerned with growing viable colonies of *P*. *destructans*, but detecting its presence by molecular methods, as well as testing for the presence of other fungi, the use of Sabouraud’s broth was deemed to be an acceptable compromise.

The bat and environmental sample that were tentatively reported as positive by PCR were shown to be negative upon confirmatory testing. The PCR that was used is not specific for *P*. *destructans* and will also detect closely related fungi [39], which was most likely the reason for these results in our study. A more specific PCR can be used to exclude false positives [55], but as the purpose of this study was primarily to determine absence or presence of the pathogen, rather than to determine its prevalence, maximal sensitivity, rather than specificity, was deemed to be the more important characteristic for initial testing. The PCR employed in our study has a sensitivity of 96% [39] and is still being used for *P*. *destructans* surveillance in North America [56].

Despite the absence of *P*. *destructans* in the samples, the fungal diversity survey showed that the genus *Pseudogymnoascus* is present in Australia, as low concentrations of *P*. *roseus* were detected on one bat. This is not surprising, as the group contains a large number of widely distributed saprophytic fungi [57]. Although it is related to *P*. *destructans* [58], *P*. *roseus* is a saprophyte found associated with soil, roots and wood [59]. It has greater saprophytic enzyme activity than *P*. *destructans*, allowing it to grow faster and utilize a broader range of substrates [60, 61], and is not known to cause disease.

A great diversity of predominantly environmental and plant-associated fungi was identified on the skin of bats. While several genera found in bat fungal surveys from Europe and North America were also detected in this study [11–14], many of the species were different possibly due to variations in cave environments and bat species. A recent sediment survey of three caves within the Naracoorte Caves National Park found a range of different fungal species [62], but none of the species found in these caves were present on the three bats that were sampled in this study. While the bats were not from the caves sampled for the environmental survey their cave was located within one kilometre of the other caves. The fungal flora of the bats’ cave is not known but bats rarely venture onto the floor of the cave resulting in minimal contact with the cave sediment.

A few fungi which, in rare circumstances or immunocompromised individuals can become pathogenic were identified. *Aspergillus flavus*, which was only found on one southern bent-winged bat from Naracoorte, is primarily a plant pathogen, but can produce aflatoxins which, when ingested, have been implicated as a cause of hepatic necrosis, and can cause chronic mucosal and systemic infections in a range of animal species [63]. While *Rhodotorula mucilaginosa* (found on eastern bent-winged bats from Christmas Hills and southern bent-winged bats from Allansford and Naracoorte) was thought to be non-pathogenic and is commonly isolated from foods and beverages, it has caused fungaemia in humans, associated with the use of intravenous catheters [64]. *Cryptococcus albidus*, found at low levels on five Christmas Hills eastern bent-winged bats, is a saprophytic yeast that has occasionally caused disease. It has been isolated from cases of meningitis, fungaemia, and pulmonary and cutaneous infections in humans [65], genital infection and keratitis in horses, and systemic disease in a dog [66]. *Cryptococcus laurentii*, found on one Christmas Hills eastern bent-winged bat, is also a saprophyte, but has caused cutaneous infection in humans [67]. *Trichosporon (Cutaneotrichosporon) cutaneum*, found on two Allansford southern bent-winged bats, has caused hair infections and onychomycosis in humans [68]. *Trichosporon (Cutaneotrichosporon) guehoae*, found on a Christmas Hills eastern bent-winged bat, was recovered from the urine of a hospitalised patient, associated with a contaminated urinary catheter [69]. *Chrysosporium* sp., found on the dead southern bent-winged bat from Portland 2 cave, has caused dermatitis and cellulitis in snakes [70]. There are, however, no reports of any of these fungal species causing disease in bats.

Bats and their environment were tested for *H*. *capsulatum*, not because of its capacity to cause disease in bats, but because of its zoonotic potential and the role bats play in its maintenance and dissemination. *Histoplasma capsulatum* has caused disease in humans in Australia [17–19], including one Victorian case that was thought to originate from Mabel Cave, near Buchan (37.5122° S, 148.1658° E), in eastern Victoria [71]. At least one of these outbreaks was associated with caves inhabited by bats of the *Miniopterus* genus (Hunt et al. 1984).

The role that other bat species may play in the maintenance and dissemination of *H*. *capsulatum* in Australia is unknown. Previous studies have detected this fungus on a large number of different bat species [21], but the ability to detect it at any given time varies. One study cultured *H*. *capsulatum* from the lungs, liver, spleen, intestines and kidneys of 50/302 Mexican free-tailed bats (*Tadarida braziliensis*) [72], while other studies failed to detect it in 108 big brown bats (*Eptesicus fuscus*), and 86 Mexican free-tailed bats, even though it could be isolated from soil contaminated with their guano [72, 73]. The reasons for these differences are unknown, but were suggested to be related to variations in the microclimate within the cave environment, and differences in air flow and bat roosting behaviour [21, 73].

While some of these studies failed to find fungi on bats even though it was recovered from their environment, the converse was the case in our study. *Histoplasma capsulatum* was identified by PCR on swabs taken directly from bats, but it was not identified in any substrate or air samples that were tested. This was unexpected, as *H*. *capsulatum* is thought to grow in guano and the surrounding cave environment [16, 19, 22], which also seems the most likely source of exposure for the bats. Previous studies have had variable success in isolation of *H*. *capsulatum* from different regions within caves when testing air and substrate samples [19, 22, 72], but none were completely negative.

Temperatures within the caves tested in our survey are generally below 20°C year round [8, 74]. The mould form of *H*. *capsulatum* grows optimally at temperatures between 22 and 29°C [19]. Therefore, it is conceivable that the temperatures in the caves surveyed are too low to allow fungal proliferation and it is only present in the substrate at very low levels. This possibility is supported by experimental work that has shown that, while *H*. *capsulatum* remained viable at 5 or 10°C, it did not grow at these temperatures [75]. The largest cave-associated outbreak of histoplasmosis in Australia, involving 16 people, occurred after exposure to Church Cave (34.3098° S, 149.9675° E) in New South Wales [19]. This cave has a mean annual temperature of 23°C [74], which is within the preferred growth temperature range of *H*. *capsulatum*. Although not statistically significant when location was taken into consideration, the trend was for bats to be more likely to be positive for *H*. *capsulatum* in the summer than the autumn, when cave temperatures are warmer and the bats are more active. This suggests that the lower environmental temperatures in these southern caves may have been responsible for its apparent absence in environmental samples, but more samples collected over the seasons would be required to confirm this.

At 35–37°C, *H*. *capsulatum* grows as a yeast [16]. While not hibernating, bats maintain a body temperature within this range [76, 77]. It is plausible that the bats were exposed to low levels of the fungal mycelium in their environment, which then morphed into the yeast form on their skin and multiplied, suggesting that they can act as an amplifying host. However, in this form the fungus is not contagious [20], which minimises the risks to the humans handling them. As further evidence of this, two of the authors (PH and LL) had blood samples collected from them at the end of the study for *H*. *capsulatum* serology (Immunodiffusion Assay, Infectious Diseases and Microbiology, Westmead Hospital, NSW), and both were found to be negative. As seroconversion takes two to six weeks [16], it might have been expected that, after more than two years of work with these bats, had the authors been exposed to the fungus, they would have seroconverted.

In conclusion, *P*. *destructans* was not found on any of the bent-winged bats sampled nor in their environment, further substantiating the view that this fungus is absent from these environments. However, as *P*. *destructans* has been identified as a potential future risk factor, should it enter Australia [8], research is required to further evaluate the vulnerability of Australian bats to WNS by gathering more information on their biology, hibernation behaviour and the climate of the caves they occupy.

Low levels of *H*. *capsulatum* were detected in most of the bat populations sampled but cave temperatures in the region sampled here appeared to be too low for mycelial growth, minimising the zoonotic risk.

Southern and eastern bent-winged bats carry a large number of fungi on their skin, mostly environmental and plant-associated. While some of these have pathogenic potential, no clinical signs suggestive of the diseases these fungi might be expected to cause have been described in these bats to date. Based on these results, fungi are unlikely to be contributing significantly to the population decline of the southern bent-winged bat. As this is the first fungal survey of bent-winged bats to be conducted in Australia, it has provided valuable baseline data about the normal fungal flora of these bats.

### Permits

Samples were collected with approval from the Faculty of Veterinary and Agricultural Science Animal Ethics Committee, University of Melbourne, Victoria (ethics approval 1513456.1), the Department of Environment, Land, Water and Planning, Victoria (permit number 0007644), the Wildlife Ethics Committee, South Australia (permit number 37/2015) and the Department of Environment, Water and Natural Resources, South Australia (permit number Q26488-1).
